# Improving Child and Adolescent Mental Health: A Bibliometric Analysis of Related Intervention Studies

**DOI:** 10.3390/ijerph21121576

**Published:** 2024-11-27

**Authors:** Gaoran Chen, Wenqi Chen, Shaojie Qi, Daniel T. L. Shek

**Affiliations:** 1 Department of Applied Social Sciences, The Hong Kong Polytechnic University, Hong Kong 999077, China; gaorchen@polyu.edu.hk; 2 Research Institute of Social Development, Southwestern University of Finance and Economics, Chengdu 610074, China; 2220303z1001@smail.swufe.edu.cn (W.C.); qishaojie@swufe.edu.cn (S.Q.)

**Keywords:** bibliometric analysis, child and adolescent mental health, knowledge production, mental health intervention

## Abstract

In response to the increase in adolescent mental health problems, related intervention research has flourished. This study examined 2258 mental health intervention studies captured by the Web of Science, focusing on their distribution, interdisciplinary collaboration, and emerging trends, using bibliometric analysis. Our findings revealed a rise in studies and enhanced collaboration across disciplines, with studies from the United States, Australia, and the United Kingdom showing high academic output, intellectual impact, and strong scientific partnerships. However, there is a noticeable Western-centrism in the research. Identifying current trends and key areas of focus offers valuable insights for future practices in child and adolescent mental health.

## 1. Introduction

The mental health status of children and adolescents is crucial for both their present psychological well-being and future health outcomes [1]. Unfortunately, many children and adolescents worldwide experience mental health issues. The interconnection between mental health and other health conditions highlights the key fact that true health cannot exist without mental well-being [2]. The World Health Organization estimates that over 10% of children and adolescents suffer from mental health problems. However, these issues frequently remain unrecognized and untreated [3]. The mental health of children and adolescents is becoming increasingly serious due to global risks such as COVID-19 and localized conflicts [4,5,6]. Moreover, significant inequalities exist in child and adolescent mental health. For instance, the highest rates of depressive symptoms were found in the Global South [7]. Access to formal psychological health services in these regions is less than one-fifth of that in high-income countries [8].

Over the past several decades, frontline mental health service workers, policymakers, and researchers have made sustained efforts to improve the psychological well-being of children and adolescents, recognizing the necessity of effective interventions [9]. Recent mental health programs have shown the effectiveness of various interventions, including digital mental health interventions, personalized interventions, rapid mental health assessment instruments, and strengths-based interventions [10,11,12,13]. Despite these efforts, resources and practices to promote mental health remain inadequate, especially in developing countries [14,15].

Governments and international organizations have prioritized improving the psychological health of children and adolescents, with numerous countries placing prevention of mental health problems on their public health and political agendas. This has resulted in more resources for mental health interventions and a rise in related research [1,16]. Social sciences, encompassing fields such as psychology, social policy, and social work, have faced criticism for their Western-centric orientation. This Western-centered approach implies that a significant portion of research is predominantly focused on Western societies. Consequently, the intervention methods developed within these disciplines often may fail to adequately consider the diverse cultural, social, and economic contexts of non-Western regions [17,18]. Henrich et al. describe this as WEIRD research to summarize research work conducted in Western, educated, industrialized, rich, and democratic countries, arguing that non-WEIRD populations have been under-researched [19]. Understanding knowledge production in interventions for child and adolescent mental health is crucial for effective and efficient improvement of mental health outcomes [20], yet our understanding remains limited. Although some studies offer overviews or systematic reviews of child and adolescent mental health interventions, they frequently lack the capacity for a quantitative analysis of knowledge development in this field [21,22,23].

Child and adolescent mental health interventions have been significant in both academic research and policy advocacy, as evidenced by the considerable number of articles published in recent years. Therefore, a systematic review based on bibliometric analysis using a large scientometric database is necessary to thoroughly examine the literature on child and adolescent mental health interventions. Bibliometric analysis employs statistical and computational techniques to explore various aspects of scientific activities, including their inputs, outputs, and processes. This method is extensively utilized to analyze knowledge structures and examine the evolution of disciplines. Additionally, it serves as a tool for evaluating scientific output and anticipating future research trends. Furthermore, bibliometric analysis plays a crucial role in mapping knowledge production and understanding epistemic politics [18,24]. Although more commonly applied in natural sciences, bibliometric analysis has gained increasing attention in social sciences in recent years [25,26,27].

This study addresses the following questions: Which countries or regions are most productive in this field? What are the recent developmental trends and hotspots in this field? How is interdisciplinary collaboration evolving? We begin by briefly introducing our research methods, followed by a presentation of our findings in three areas: countries/regions, hotspots and trends, and interdisciplinary collaboration. We conclude with a discussion on knowledge production and its implications for child and adolescent mental health in practice. This review is very important because it gives us a picture of the current research gaps and the future directions regarding child and adolescent mental health intervention programs.

## 2. Materials and Methods

### 2.1. Data Filtering and Data Cleaning

**Figure 1:**
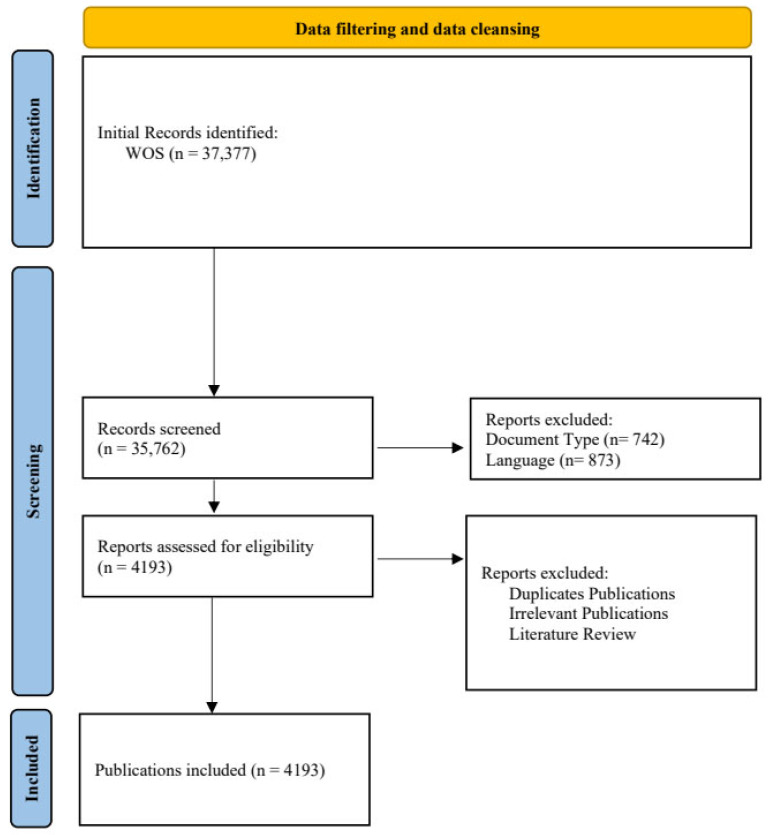
Article selection procedure. The temporal parameters of our study are from 1 January 1991 to 20 April 2024 (The earliest literature available in the WoS Core Collection dates back to 1991).

We utilized the Web of Science (WoS) database, widely considered ideal for scientometric analysis due to its extensive literature and rich publication data [24,28]. The data filtering and cleansing process involved several steps. First, we used the search formula “Topic = (mental health)” and “Topic = (child or adolescent) and (intervention or plan or program or project)” to search within the WoS core collection. Second, document types other than reviews and articles lacking essential analytical information, such as references, countries, and keywords, were included. Additionally, as current analytical tools cannot process multilingual literature simultaneously, we searched further by applying the following criteria: “document type = (article + review)” and “language = (English)”. Third, to ensure the accuracy of the included literature, non-intervention studies, duplicate studies, and review articles were excluded. This selection process was conducted independently by two authors adhering to the PRISMA statement guidelines [29] to maintain consistency in the results. This process resulted in the inclusion of 2258 publications for scientometric analysis. Figure 1 shows the detailed information on our data filtering process.

### 2.2. Indicators

Number of publications (NP) refers to the total publications produced by each entity.

Number of citations (NC) refers to the number of citations received by papers published by different production entities.

Betweenness Centrality (BC) is a crucial metric for assessing the significance of contributors within networks. Widely utilized in network analysis, BC quantifies a node’s centrality by evaluating its role in facilitating communication across the network [30]. Specifically, BC is determined by calculating the number of shortest paths that traverse a given node, connecting all vertices within the network [31]. The BC is calculated as follows:

(1) $${\mathrm{BC}}_{i}=\sum_{s\neq i\neq t}\frac{n_{\mathrm{st}}^{i}}{g_{\mathrm{st}}}$$

In this context, $g_{\mathrm{st}}$ represents the total number of shortest paths from node s to node t, among which $n_{\mathrm{st}}^{i}$ is the number of shortest paths passing through node i. A higher BC value indicates that the contributor plays a more significant “bridge” role in scientific research networks. BC was calculated after collaboration network analysis using CiteSpace (version 6.4.R1) [32].

The Socio-demographic Index (SDI) assesses the level of development of a country or region, calculated from indicators such as average income, education level, and fertility rate [33]. This study utilized SDI data from the Global Burden of Disease (GBD) database. For overall analysis, SDI values represent the average from 1991 to 2019. For period-specific analysis, SDI values are averaged for the periods 1991–2000, 2001–2010, and 2011–2019, as GBD does not update SDI data beyond 2019.

### 2.3. Data Analytical Plan

First, we analyzed temporal trends in total publications and citations. Second, we conducted spatial analysis of publication and citation numbers and examined scientific cooperation between countries and regions. The Spearman correlation was used to analyze the relationships between research activity quantity, impact, collaboration, and SDI. Third, we analyzed interdisciplinary collaboration and its changes through network analysis [24]. Fourth, we used co-citation network analysis and burst detection in CiteSpace to identify hotspots and research trends in child and adolescent mental health interventions.

Co-citation network analysis is a widely utilized method in bibliometric research [21]. This method constructs a complex network among scholarly works within a particular field by examining the relationships between citing and cited literature. The underlying premise is that citing and cited works often share a close relationship and similar academic background [34]. Through the co-citation network, this analysis aims to identify significant literature within the field, thereby enhancing our understanding of the intellectual structure and influential works in the domain. Specifically, the BC value was used to identify a “bridge” literature relevant to research hotspots in the co-citation network. Burst detection identifies research trends and frontiers by detecting significant changes in citation frequency [23,35]. We utilized continuous bursts from recent literature to identify emerging research trends.

## 3. Results

### 3.1. Annual Publishing Trend

**Figure 2:**
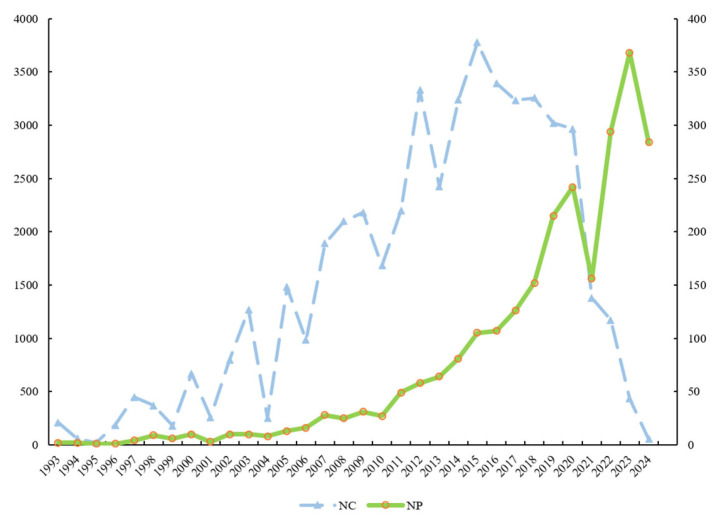
Annual publishing and cited trend of the countries/regions from 1991 to 2024. The right ordinate represents the number of articles published, while the left ordinate indicates the number of articles cited. NP = number of publications. NC = number of citations.

Figure 2 illustrates a significant increase in publications on child and adolescent mental health interventions over the past three decades. This growth became particularly pronounced after 2010, with the annual number of publications exceeding 100 after 2015 and reaching 368 in 2023 alone, yet NP declined significantly in 2021, probably because of COVID-19 pandemic, and then the NP issued rebounded. The NC for research in this area also saw a significant increase until 2015, suggesting a rapid rise in the impact of these studies. The decline in citations post-2015 may be attributed to the time lag in the dissemination of academic impact, necessitating a longer period to effectively assess changes in research influence.

### 3.2. Spatial Analysis of Knowledge Production

This section examines the geographical distribution of knowledge production in child and adolescent mental well-being interventions, focusing on NP, NC, and academic collaborations across countries and regions.

**Figure 3:**
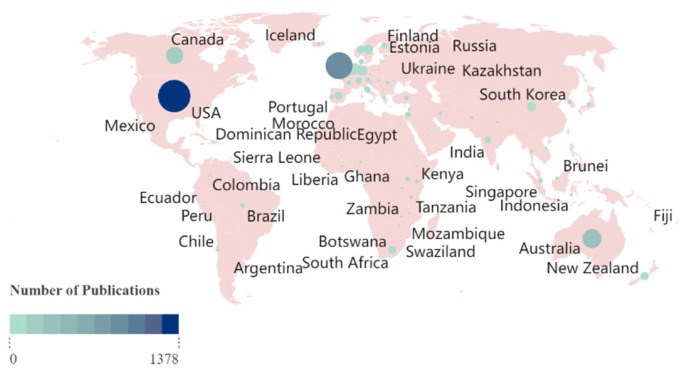
Publications for the countries/regions from 1991 to 2024. Each node represents a country or region, with its size and color indicating the number of publications. Larger nodes and deeper colors signify a higher volume of literature published from these countries or regions.

Many social scientists argue that there is an anglophone dominance in social science publications [17,19,24,36], a notion partly supported by our data. From 1991 to 2024, researchers from 101 countries and regions published work in this field, covering almost all parts of the world. However, the distribution of these publications is uneven, as shown in Figure 3. The United States (*n* = 1080), the United Kingdom (*n* = 477), and Australia (*n* = 389) significantly outpaced all other countries. Although non-Western countries like China, India, and South Africa have recently played vital roles in producing knowledge on child and adolescent mental health interventions, Western countries still dominate the overall landscape.

**Figure 4:**
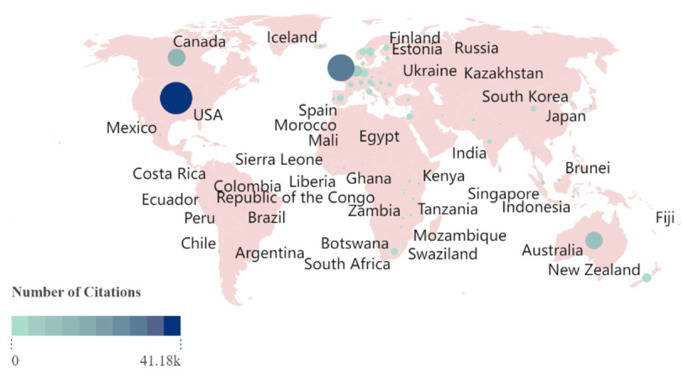
Citations for the countries/regions from 1991 to 2024. Each node represents a country or region, with its size and color indicating the number of citations from that area. Larger nodes and deeper colors signify a higher number of citations.

**Figure 5:**
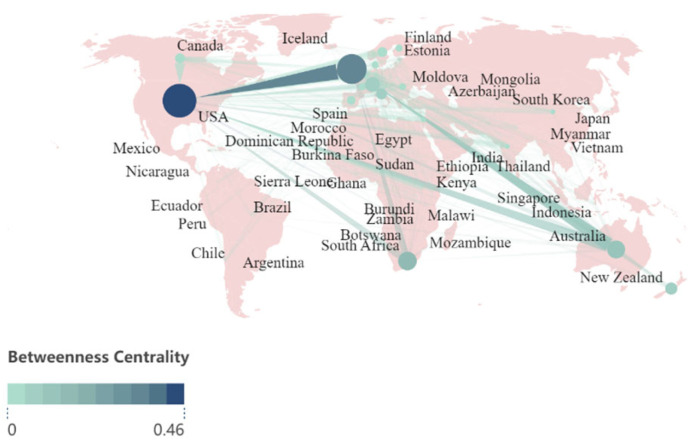
Collaborations of the countries/regions from 1991 to 2024. In this figure, each node symbolizes a specific country or region. The size and color of the nodes are indicative of the significance of each country or region within the collaborative networks. Specifically, larger nodes or those with deeper colors represent countries or regions that play a more substantial role as “bridges” in research cooperation. The connections between nodes signify collaborative relationships between different countries or regions.

Similar patterns are observed in the number of citations (Figure 4). The United States, Australia, and United Kingdom and are leaders in academic impact, with the United States receiving more citations than the United Kingdom and Australia together. Additionally, there is a high level of scientific cooperation among these three countries (Figure 5). Notably, South Africa, a low-income country, maintains close scientific cooperation with United States, Australia, and United Kingdom. This suggests a shift in research interest towards less developed regions, where non-Western countries are emerging as significant sources of knowledge production.

A Spearman correlation analysis was conducted to explore the relationships among NP, NC, research collaborations, and the development status of countries or regions. The Spearman correlation coefficients for the NP, NC, and BC were 0.528, 0.547, and 0.607, respectively, indicating that higher development levels are associated with greater productivity, research impact, and closer research collaboration. Countries with high SDI values are concentrated in North America, Western Europe, and Oceania, consistent with previous findings.

**Figure 6:**
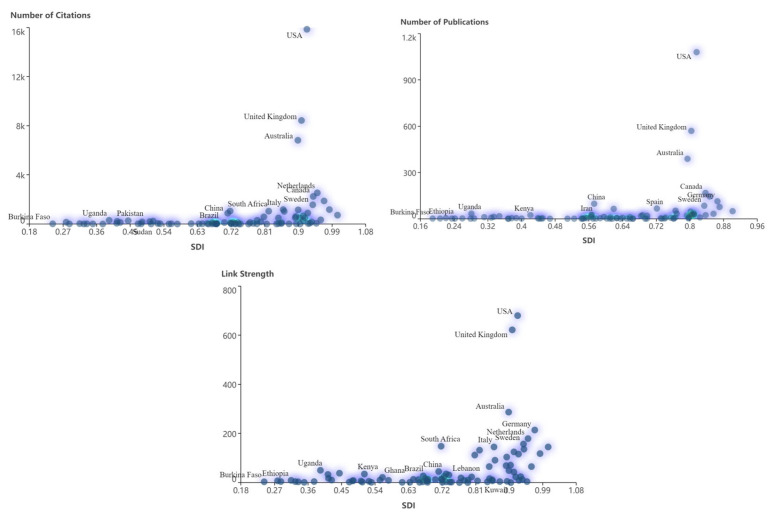
The relationship between number of publications, number of citations, the link strength for the countries/regions, and SDI from 1991 to 2024.

Figure 6 further visualizes the relationship between SDI and the NP, NC, and research collaborations. We can find United States, Australia, and United Kingdom as outliers, leading in productivity, impact, and research collaboration while belonging to the high SDI countries. Thus, the United States, Australia, and United Kingdom are the most important and productive triangle in child and adolescent mental health intervention with their notable productivity, wide-ranging influence, and intensive collaborations.

### 3.3. Discipline Collaboration Network Analysis

**Figure 7:**
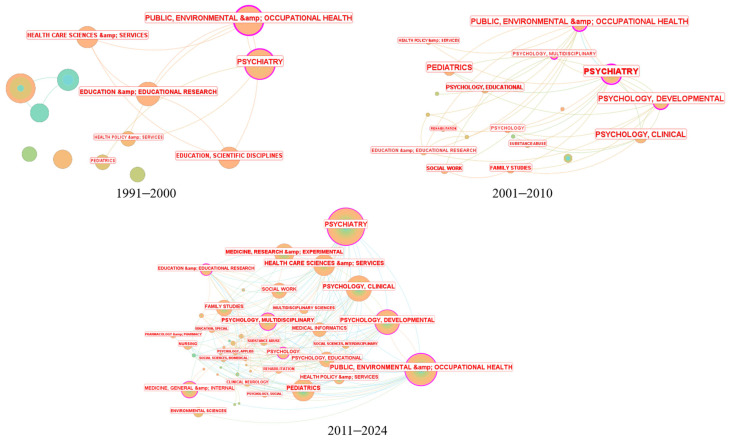
Interdisciplinary cooperation network. Each node indicates disciplines, where larger nodes represent a higher number of published papers. Edges between nodes indicate collaborative relationships between disciplines.

To understand changes in interdisciplinary collaboration, we analyzed the status of interdisciplinary collaboration in three periods: 1991–2000, 2001–2010, and 2011–2024 (Figure 7).

In the early stage (1991–2000), the number of participating disciplines and interdisciplinary collaborations was relatively small, with Psychiatry and Public Environment and Occupational Health being dominant. The period 2001–2010 saw an increase in participating disciplines and collaborations, including sub-disciplines like Developmental Psychology and Clinical Psychology. The period 2011–2024 showed a significant increase in participating disciplines and stronger interdisciplinary cooperation. The involvement of fields such as Zoology and Music suggests an expansion of research perspectives in child and adolescent mental health interventions.

### 3.4. Co-Citation Network Analysis and Burst Detection

**Figure 8:**
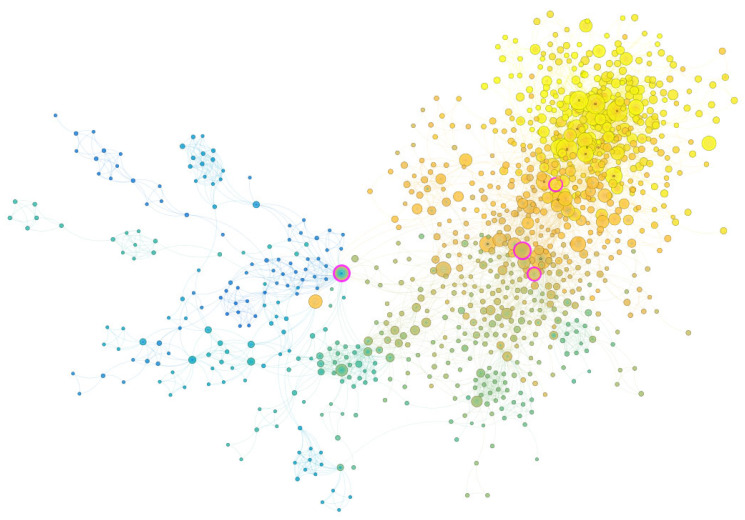
Co-citation network of child and adolescent mental health interventions. Each node represents one cited reference, and each edge indicates the co-citation relationship. The color represents the date of publication: Yellow indicates literature that is newly published, and green and purple indicate literature published in earlier years.

We used co-citation analysis to reveal the overall structure of the knowledge domain of child and adolescent mental health intervention research in a broader context. The nodes in the co-citation network depict the underlying structure of the literature in this field. Figure 8 depicts the co-citation network in child and adolescent mental health intervention research, where each node represents a cited reference, and each edge signifies a co-citation relationship. The color represents the publication date: yellow for newly published literature and green and purple for older literature. BC was computed to identify key literature in the network. Table 1 lists the 15 papers with the highest BC values, reflecting research hotspots in this field.

First, the book, *Diagnostic and statistical manual of mental disorders*, edited by the American Psychiatric Association and published in 2011 had the highest BC, showing that it served as a crucial node linking multiple studies. Second, the study with the second highest BC was *Psychological and educational interventions for preventing depression in children and adolescents* (author: Merry et al., published in 2011), which reviewed fifty-three studies on intervention for depression among children and adolescents and found that many interventions applied cognitive behavioral therapy and emphasized the importance of placebo in future interventions .

Table 2 lists the 15 papers that will remain of high interest until 2024, analyzed by burst detection, and reflected the research trends in this field. The paper, *Global Prevalence of Depressive and Anxiety Symptoms in Children and Adolescents During COVID-19: A Meta-analysis,* published by Racine et al. had the strongest of burst strength. This study, encompassing 80,879 children and adolescents across 29 studies, found that the global prevalence of depression and anxiety symptoms among this group has doubled during COVID-19, particularly among girls . The study titled “*School-based depression and anxiety prevention programs for young people: A systematic review and meta-analysis*” by Werner-Seidler et al., published in 2016, demonstrated the second-strongest burst strength in its findings. This suggests that refining school-based prevention programs holds significant potential for reducing the mental health burden among students .

## 4. Discussion

### 4.1. Interdisciplinary Collaboration

Mental health issues such as depression have complex causes that encompass biological, social, economic, and cultural factors [64,65,66,67]. To improve the effectiveness of psychological well-being interventions for children and adolescents, interdisciplinary collaboration is increasingly advocated [50,68]. Although not all intervention programs currently involve multiple disciplines, interdisciplinary collaboration is becoming a trend in this research area as shown in the present review. For instance, Hoagwood and colleagues combined Cognitive Behavioral Therapy (CBT) with adaptive riding to develop the RiA intervention course, which effectively reduced mild to moderate anxiety in adolescents aged 6–17 years [69]. Another example is Rap Music Therapy (RMT), an interdisciplinary collaboration between psychology and music, which has been shown to regulate adolescents’ emotions effectively [70].

### 4.2. Inequality in Knowledge Production

Examining knowledge production in child and adolescent mental health intervention necessitates a reflexive approach. Children and adolescents in global south counties face more severe mental health issues [7], compared to the global average, yet progress in intervention research in these countries has been relatively limited. Undoubtedly, this is not conducive to alleviating the overall mental well-being plight of children and adolescents globally. In general, research on child and adolescent mental health interventions is rife with Western-centrism. Non-Western countries and researchers face the following dilemmas: firstly, non-Western populations are understudied [19,71]. Secondly, Western-centrism implies not only academic hierarchies in terms of knowledge producers and sites of production, but also in terms of the language used. Non-English-speaking scholars are under pressure to become multilinguals and citizens of the world; they must learn and follow the rules of Western scholarship in their enquiry and production of knowledge and seek to publish in English [72]. Thirdly, there exists a clear “academic dependency” where non-Western social scientists rely on Western theorists, creating a global intellectual division of labor. In this arrangement, Western scholars primarily engage in theorizing, while others focus on data collection [25,73,74]. Despite these challenges, non-Western researchers are striving to challenge Western-centrism and promote localized intervention research. The P.A.T.H.S. and Tin Ka Ping P.A.T.H.S. programs are notable examples, combining Chinese Confucian cultural values with positive youth development theories. These programs have been implemented in Hong Kong and mainland China for over a decade, benefiting over 300,000 students in Hong Kong alone [68,75]. Evaluations indicate that these programs enhance participants’ well-being and reduce mental health risks [76,77]. The Tin Ka Ping P.A.T.H.S. program was originally intended for urban schools in Chinese mainland. Use of the Tin Ka Ping P.A.T.H.S. in rural area has also shown positive outcomes in poor mountainous areas [78]. The P.A.T.H.S. program was the only Chinese initiative recognized globally for effectively improving adolescents’ mental health [79]. Despite these successes, more work is needed to advance research on child and adolescent mental health interventions and amplify non-Western voices.

There are three possible factors contributing to the inequality of studies between the West and non-Western worlds. First, more studies in the West can simply be regarded as a reflection of the more serious adolescent mental health problems in the West. Such higher figures may also be due to more sensitive catchment (such as more professionals for school children) and higher stress in the urban and industrialized Western world [80,81,82,83]. Second, more research in the West may be a reflection of the greater emphasis these countries place on the welfare of children and adolescents, as reflected in the more holistic and integrated policies on children and adolescent mental health. In non-Western countries, child mental health policies are rather disjointed [14,15,81]. Finally, the abundance of research in Western societies is simply due to the fact that there are more leading universities in these places. These are exciting possibilities for future research.

### 4.3. Hotspots and Trends

Our study used co-citation analysis and burst detection to identify key topics in child and adolescent mental health interventions. The emerging hotspots and trends include digital interventions, theoretical and practical aspects of mental health interventions, and research methods.

Digital or internet interventions use technology to expand face-to-face intervention scenarios and tools [51,84]. These interventions have gained popularity due to their cost-effectiveness and efficiency [42,61]. During the COVID-19 pandemic, digital interventions were essential for addressing severe mental wellbeing issues in children and adolescents when face-to-face interventions were not possible [85]. Evidence suggests that digital interventions effectively alleviate psychosocial problems at the population level [86,87]. Since November 2022 when ChatGPT, an AI assistant developed by Open AI, was generated, AI has seen a surge in development, and we are moving towards an era of highly developed AI [88]. For child and adolescent psychological digital intervention, AI brings new opportunities [89]. How to rationally use the advantages of the big model to develop new digital intervention products is the question we must answer now and for a long time to come. Obviously, evidence-based intervention plays a crucial role in validating such interventions. Nonetheless, in today’s highly digitized world, we face an equally stark digital divide [90]. Making the fruits of digital interventions more equally available to children and adolescents globally is another issue that we need to face. Again, this observation echoes the use of an interdisciplinary approach to address child and adolescent mental health issues.

CBT addresses negative emotions by influencing cognition and behavior through various psychological strategies [91]. Empirical evidence supports CBT’s effectiveness in improving adolescent mental health [92,93]. The National Institute for Health and Clinical Excellence (NICE) recommends CBT as the primary treatment for depression in children and adolescents [94]. Recent advancements in digital interventions and interdisciplinary approaches have enriched traditional CBT [95]. However, the high cost of CBT has led to calls for reducing treatment costs or developing alternative treatments [42]. School-based interventions and mental health prevention programs are recognized as cost-effective alternatives [40,47,49]. In addition, CBT faces the problem of cultural sensitivity; for example, the basic extent of CBT may conflict with some religious concepts [96].

In addition, interventions based on Positive Youth Development (PYD) theory are experiencing rapid growth. As a theoretical perspective, PYD has spawned a range of theoretical models, intervention strategies, and successes in the field of practice. Unlike the ‘deficit’ perspective, which emphasizes the problems that adolescents exhibit in their development, PYD emphasizes adolescents’ talents, strengths, interests, and developmental potential, viewing them as valuable resources for social development [97]. PYD focuses on the holistic development of adolescents rather than focusing on just one issue in adolescent development, as well as the environment in which adolescents live and how they grow, learn, and change [98]. Inspired by PYD, a large number of intervention projects have been implemented within the Social Emotional Learning (SEL) framework. The result of meta-analysis found that SEL interventions can effectively improve mental well-being among children and adolescents [41,99]. Although a large number of PYD intervention projects have been or are being implemented globally, the vast majority of projects are in WEIRD countries [17].

The validity of social sciences has long been questioned when compared to natural sciences [100]. The concept of evidence-based medicine has permeated social sciences, emphasizing the importance of evidence-based approaches [101]. Evaluating intervention effects and designing normative intervention programs are crucial for evidence-based social science. Randomized controlled trials (RCTs) and meta-analysis techniques have evolved, allowing for more objective assessments of intervention programs [102,103]. RCTs are the gold standard for evaluating the effectiveness of interventions and are extensively used in child and adolescent mental health [104,105,106]. Most intervention studies have been conducted in a limited number of sites and populations, and it has become an important issue to integrate the results of multiple studies and to comprehensively assess the effectiveness of intervention methods. Meta-analysis is the key method for synthesizing the results of existing intervention studies [107]. A growing number of child and adolescent intervention researchers are conducting or considering meta-analysis [108]. In fact, a large number of intervention studies are inadequate in terms of the rationality and reproducibility of their intervention programs. To address this problem some guidelines and statements have been advocated [109]. For example, the SPIRIT Group, which has launched relevant initiatives and provided guidance to help improve the integrity and quality of intervention programs [44]. There are many more such examples. Child and adolescent mental health intervention research is moving towards a more standardized future.

The present study highlights that although child and adolescent mental health intervention research has been fruitful, challenging Western-centrism in research, a more rational allocation of global research resources and increased interdisciplinary collaboration are essential to effectively address global child and adolescent mental health issues.

For frontline clinicians, it is essential to integrate the latest advances in digital interventions, CBT, and PYD interventions into child and adolescent mental health practice. We advocate for the use of evidence-based scientific methods to incorporate these research findings effectively. Meanwhile, researchers, particularly those from non-Western contexts, should focus on strengthening localized mental health interventions for children and adolescents with reference to local, national, and international contexts. In particular, this approach should consider the cultural influences and practical implementation issues of these interventions within the local context during the research process. As an example, one can look at the application of the positive youth development approach in different Chinese contexts is the Tin Ka Ping P.A.T.H.S. project [110].

### 4.4. Limitations

Our study shares limitations common to bibliometric analyses. First, it is limited to English-language and published papers. Future studies could expand data sources to include various linguistic backgrounds, conference articles, and books. Second, our research primarily relies on bibliographic records, making it difficult to focus on practical research processes and the rich information behind the literature. Third, the search could be enhanced by expanding beyond the term “mental health” to include related terms such as “depression”, “anxiety”, and “psychological problems”, which some researchers might use. Obviously, future studies should be conducted with more refined terms related to mental health, such as “depression”, “anxiety disorder” and “substance abuse”. Finally, our research is limited to evaluating published literature, making it challenging to objectively assess ongoing intervention projects. These areas require continued attention.

## 5. Conclusions

This research provides a bibliometric analysis of child and adolescent mental health interventions (1991–2024) to demonstrate the patterns and politics of knowledge production in this field. Our findings generate three observations. Firstly, academic work on child and adolescent mental health intervention has rapidly increased since 1991. Secondly, interdisciplinary collaboration is becoming increasingly popular in child and adolescent mental health intervention. Thirdly, research on child and adolescent mental health interventions in the English-speaking countries, including the United States, Australia, and the United Kingdom demonstrates remarkable academic productivity, significant intellectual influence, and strong scientific collaboration, establishing a leading role in this field. Despite its growing importance in the field, non-Western countries, particularly China, appear to be lagging, with a significant gap still evident.

## Figures and Tables

**Table 1:** Top 15 reference with the highest BC.

No.	Author	Year	BC	Reference
1	American Psychiatric Association [37]	2011	0.28	Diagnostic and statistical manual of mental disorders
2	Merry et al. [38]	2011	0.12	Psychological and educational interventions for preventing depression in children and adolescents
3	Calear et al. [39]	2010	0.11	Systematic review of school-based prevention and early intervention programs for depression
4	Stockings et al. [40]	2016	0.11	Preventing depression and anxiety in young people: a review of the joint efficacy of universal, selective and indicated prevention
5	Durlak et al. [41]	2011	0.09	The impact of enhancing students’ social and emotional learning: a meta-analysis of school-based universal interventions
6	Ebert et al. [42]	2015	0.07	Internet and computer-based cognitive behavioral therapy for anxiety and depression in youth: a meta-analysis of randomized controlled outcome trials
7	Richardson et al. [43]	2010	0.06	Computerised cognitive behavioural therapy for the prevention and treatment of depression and anxiety in children and adolescents: a systematic review
8	Chan et al. [44]	2013	0.06	SPIRIT 2013 explanation and elaboration: guidance for protocols of clinical trials
9	Eyberg et al. [45]	2008	0.06	Evidence-based psychosocial treatments for children and adolescents with disruptive behavior
10	Ehntholt et al. [46]	2005	0.06	School-based Cognitive-Behavioural Therapy Group Intervention for Refugee Children who have Experienced War-related Trauma
11	Corrieri et al. [47]	2014	0.05	School-based prevention programs for depression and anxiety in adolescence: a systematic review
12	Kieling et al. [48]	2011	0.04	Child and adolescent mental health worldwide: evidence for action
13	Fisak et al. [49]	2011	0.04	The prevention of child and adolescent anxiety: a meta-analytic review
14	Kazdin et al. [50]	2011	0.04	Rebooting Psychotherapy Research and Practice to Reduce the Burden of Mental Illness
15	Andersson et al. [51]	2009	0.04	Internet-based and other computerized psychological treatments for adult depression: a meta-analysis

**Table 2:** Top 15 reference with the highest the strongest citation bursts still in 2024.

No.	Author	Year	Begin	End	Strength	Reference
1	Racine et al. [52]	2021	2022	2024	14.09	Global Prevalence of Depressive and Anxiety Symptoms in Children and Adolescents During COVID-19: A Meta-analysis
2	Werner-Seidler et al. [53]	2017	2020	2024	8.88	School-based depression and anxiety prevention programs for young people: A systematic review and meta-analysis
3	Weisz et al. [54]	2017	2020	2024	8.56	What five decades of research tells us about the effects of youth psychological therapy: A multilevel meta-analysis and implications for science and practice
4	Skivington et al. [55]	2021	2022	2024	8.34	A new framework for developing and evaluating complex interventions: update of Medical Research Council guidance
5	Polanczyk et al. [56]	2015	2021	2024	8.31	Annual Research Review: A meta-analysis of the worldwide prevalence of mental disorders in children and adolescents
6	Patel et al. [16]	2018	2021	2024	8.02	The Lancet Commission on global mental health and sustainable development
7	Solmi et al. [57]	2022	2022	2024	7.96	Age at onset of mental disorders worldwide: large-scale meta-analysis of 192 epidemiological studies
8	Weiner et al. [58]	2017	2021	2024	7.91	Psychometric assessment of three newly developed implementation outcome measures
9	Erskine et al. [59]	2015	2020	2024	7.81	A heavy burden on young minds: the global burden of mental and substance use disorders in children and youth
10	Loades et al. [5]	2020	2021	2024	7.51	Rapid Systematic Review: The Impact of Social Isolation and Loneliness on the Mental Health of Children and Adolescents in the Context of COVID-19
11	Taylor et al. [60]	2017	2021	2024	7.49	Promoting Positive Youth Development Through School-Based Social and Emotional Learning Interventions: A Meta-Analysis of Follow-Up Effects
12	Liverpool et al. [61]	2020	2021	2024	7.38	Engaging Children and Young People in Digital Mental Health Interventions: Systematic Review of Modes of Delivery, Facilitators, and Barriers
13	Patton et al. [1]	2016	2021	2024	7.38	Our future: a Lancet commission on adolescent health and wellbeing
14	Schleider et al. [62]	2018	2020	2024	6.86	A single-session growth mindset intervention for adolescent anxiety and depression: 9-month outcomes of a randomized trial
15	Schleider et al. [63]	2017	2020	2024	6.86	Little Treatments, Promising Effects? Meta-Analysis of Single-Session Interventions for Youth Psychiatric Problems

## Data Availability

The data that support the findings of this study are available from Web of Science (WoS) at https://www.webofscience.com, accessed on 27 June 2024.

## References

[1] Patton G.C., Sawyer S.M., Santelli J.S., Ross D.A., Afifi R., Allen N.B., Arora M., Azzopardi P., Baldwin W., Bonell C. (2016). Our Future: A Lancet Commission on Adolescent Health and Wellbeing. Lancet.

[2] Prince M., Patel V., Saxena S., Maj M., Maselko J., Phillips M.R., Rahman A. (2007). Global Mental Health 1 No Health without Mental Health. Lancet.

[3] Mental Health of Adolescents. https://www.who.int/news-room/fact-sheets/detail/adolescent-mental-health.

[4] Liang L., Ren H., Cao R., Hu Y., Qin Z., Li C., Mei S. (2020). The Effect of COVID-19 on Youth Mental Health. Psychiatr. Q..

[5] Loades M.E., Chatburn E., Higson-Sweeney N., Reynolds S., Shafran R., Brigden A., Linney C., McManus M.N., Borwick C., Crawley E. (2020). Rapid Systematic Review: The Impact of Social Isolation and Loneliness on the Mental Health of Children and Adolescents in the Context of COVID-19. J. Am. Acad. Child Adolesc. Psychiatry.

[6] Peltonen K. (2024). Children and War—Vulnerability and Resilience. Eur. J. Dev. Psychol..

[7] Shorey S., Ng E.D., Wong C.H.J. (2022). Global Prevalence of Depression and Elevated Depressive Symptoms among Adolescents: A Systematic Review and Meta-analysis. Br. J. Clin. Psychol..

[8] WHO (2022). World Mental Health Report Transforming Mental Health for All.

[9] Das J.K., Salam R.A., Lassi Z.S., Khan M.N., Mahmood W., Patel V., Bhutta Z.A. (2016). Interventions for Adolescent Mental Health: An Overview of Systematic Reviews. J. Adolesc. Health.

[10] Ng M.Y., Weisz J.R. (2016). Annual Research Review: Building a Science of Personalized Intervention for Youth Mental Health. Child Psychol. Psychiatry.

[11] Curran T., Wexler L. (2017). School-Based Positive Youth Development: A Systematic Review of the Literature. J. Sch. Health.

[12] Hollis C., Falconer C.J., Martin J.L., Whittington C., Stockton S., Glazebrook C., Davies E.B. (2017). Annual Research Review: Digital Health Interventions for Children and Young People with Mental Health Problems—A Systematic and Meta-review. Child Psychol. Psychiatry.

[13] Wainberg M.L., Scorza P., Shultz J.M., Helpman L., Mootz J.J., Johnson K.A., Neria Y., Bradford J.-M.E., Oquendo M.A., Arbuckle M.R. (2017). Challenges and Opportunities in Global Mental Health: A Research-to-Practice Perspective. Curr. Psychiatry Rep..

[14] Patel V. (2007). Mental Health in Low- and Middle-Income Countries. Br. Med. Bull..

[15] Patel V., Kieling C., Maulik P.K., Divan G. (2013). Improving Access to Care for Children with Mental Disorders: A Global Perspective. Arch. Dis. Child..

[16] Patel V., Saxena S., Lund C., Thornicroft G., Baingana F., Bolton P., Chisholm D., Collins P.Y., Cooper J.L., Eaton J. (2018). The Lancet Commission on Global Mental Health and Sustainable Development. Lancet.

[17] Hendriks T., Warren M.A., Schotanus-Dijkstra M., Hassankhan A., Graafsma T., Bohlmeijer E., De Jong J. (2019). How WEIRD Are Positive Psychology Interventions? A Bibliometric Analysis of Randomized Controlled Trials on the Science of Well-Being. J. Posit. Psychol..

[18] Qi S., Hua F., Zhou Z., Shek D.T.L. (2022). Trends of Positive Youth Development Publications (1995–2020): A Scientometric Review. Appl. Res. Qual. Life.

[19] Henrich J., Heine S.J., Norenzayan A. (2010). The Weirdest People in the World?. Behav. Brain Sci..

[20] Cai Z., Chang Q., Yip P.S.F. (2020). A Scientometric Analysis of Suicide Research: 1990–2018. J. Affect. Disord..

[21] Chen C. (2017). Science Mapping: A Systematic Review of the Literature. J. Data Inf. Sci..

[22] Huang J., Gates A.J., Sinatra R., Barabási A.-L. (2020). Historical Comparison of Gender Inequality in Scientific Careers across Countries and Disciplines. Proc. Natl. Acad. Sci. USA.

[23] Donthu N., Kumar S., Mukherjee D., Pandey N., Lim W.M. (2021). How to Conduct a Bibliometric Analysis: An Overview and Guidelines. J. Bus. Res..

[24] Luo M., Chen G., He Q., Qi S. (2022). Knowledge Production and Epistemic Politics: A Scientometric Review of Chinese Sexuality Studies in English-Language Academia. Chin. J. Sociol..

[25] Sweileh W.M., Wickramage K., Pottie K., Hui C., Roberts B., Sawalha A.F., Zyoud S.H. (2018). Bibliometric Analysis of Global Migration Health Research in Peer-Reviewed Literature (2000–2016). BMC Public Health.

[26] Sooryamoorthy R. (2020). Scientometrics for the Study of Sociology. Int. Sociol..

[27] Cataldo R., Alaimo L.S., Grassia M.G., Maggino F. (2024). How Subjective Well-Being Evolved Over Time: Insights from Bibliometric and Text Mining Analyses. Appl. Res. Qual. Life.

[28] Van Leeuwen T. (2006). The Application of Bibliometric Analyses in the Evaluation of Social Science Research. Who Benefits from It, and Why It Is Still Feasible. Scientometrics.

[29] Page M.J., McKenzie J.E., Bossuyt P.M., Boutron I., Hoffmann T.C., Mulrow C.D., Shamseer L., Tetzlaff J.M., Akl E.A., Brennan S.E. (2021). The PRISMA 2020statement: An updated guideline for reporting systematic reviews. BMJ.

[30] Leydesdorff L. (2007). Betweenness Centrality as an Indicator of the Interdisciplinarity of Scientific Journals. J. Am. Soc. Inf. Sci..

[31] Qian G. (2014). Scientometric Sorting by Importance for Literatures on Life Cycle Assessments and Some Related Methodological Discussions. Int. J. Life Cycle Assess.

[32] Chen C. (2004). Searching for Intellectual Turning Points: Progressive Knowledge Domain Visualization. Proc. Natl. Acad. Sci. USA.

[33] Haagsma J.A., James S.L., Castle C.D., Dingels Z.V., Fox J.T., Hamilton E.B., Liu Z., Lucchesi L.R., Roberts N.L.S., Sylte D.O. (2020). Burden of Injury along the Development Spectrum: Associations between the Socio-Demographic Index and Disability-Adjusted Life Year Estimates from the Global Burden of Disease Study 2017. Inj. Prev..

[34] Small H. (1973). Co-citation in the Scientific Literature: A New Measure of the Relationship between Two Documents. J. Am. Soc. Inf. Sci..

[35] Kleinberg J. (2003). Bursty and Hierarchical Structure in Streams. Data Min. Knowl. Discov..

[36] Garcia Ramon M.D., Simonsen K., Vaiou D. (2006). Guest Editorial: Does Anglophone Hegemony Permeate Gend. Place Culture?. Gend. Place Cult..

[37] American Psychiatric Association (2013). Diagnostic and Statistical Manual of Mental Disorders.

[38] Merry S.N., Hetrick S.E., Cox G.R., Brudevold-Iversen T., Bir J.J., McDowell H., The Cochrane Collaboration (2011). Psychological and Educational Interventions for Preventing Depression in Children and Adolescents. Cochrane Database of Systematic Reviews.

[39] Calear A.L., Christensen H. (2010). Systematic Review of School-based Prevention and Early Intervention Programs for Depression. J. Adolesc..

[40] Stockings E.A., Degenhardt L., Dobbins T., Lee Y.Y., Erskine H.E., Whiteford H.A., Patton G. (2016). Preventing Depression and Anxiety in Young People: A Review of the Joint Efficacy of Universal, Selective and Indicated Prevention. Psychol. Med..

[41] Durlak J.A., Weissberg R.P., Dymnicki A.B., Taylor R.D., Schellinger K.B. (2011). The Impact of Enhancing Students’ Social and Emotional Learning: A Meta-Analysis of School-Based Universal Interventions. Child Dev..

[42] Ebert D.D., Zarski A.-C., Christensen H., Stikkelbroek Y., Cuijpers P., Berking M., Riper H. (2015). Internet and Computer-Based Cognitive Behavioral Therapy for Anxiety and Depression in Youth: A Meta-Analysis of Randomized Controlled Outcome Trials. PLoS ONE.

[43] Richardson T., Stallard P., Velleman S. (2010). Computerised Cognitive Behavioural Therapy for the Prevention and Treatment of Depression and Anxiety in Children and Adolescents: A Systematic Review. Clin. Child Fam. Psychol. Rev..

[44] Chan A.-W., Tetzlaff J.M., Gotzsche P.C., Altman D.G., Mann H., Berlin J.A., Dickersin K., Hrobjartsson A., Schulz K.F., Parulekar W.R. (2013). SPIRIT 2013 Explanation and Elaboration: Guidance for Protocols of Clinical Trials. BMJ.

[45] Eyberg S.M., Nelson M.M., Boggs S.R. (2008). Evidence-Based Psychosocial Treatments for Children and Adolescents with Disruptive Behavior. J. Clin. Child Adolesc. Psychol..

[46] Ehntholt K.A., Smith P.A., Yule W. (2005). School-Based Cognitive-Behavioural Therapy Group Intervention for Refugee Children Who Have Experienced War-Related Trauma. Clin. Child Psychol. Psychiatry.

[47] Corrieri S., Heider D., Conrad I., Blume A., Konig H.-H., Riedel-Heller S.G. (2014). School-Based Prevention Programs for Depression and Anxiety in Adolescence: A Systematic Review. Health Promot. Int..

[48] Kieling C., Baker-Henningham H., Belfer M., Conti G., Ertem I., Omigbodun O., Rohde L.A., Srinath S., Ulkuer N., Rahman A. (2011). Child and Adolescent Mental Health Worldwide: Evidence for Action. Lancet.

[49] Fisak B.J., Richard D., Mann A. (2011). The Prevention of Child and Adolescent Anxiety: A Meta-Analytic Review. Prev. Sci..

[50] Kazdin A.E., Blase S.L. (2011). Rebooting Psychotherapy Research and Practice to Reduce the Burden of Mental Illness. Perspect. Psychol. Sci..

[51] Andersson G., Cuijpers P. (2009). Internet-Based and Other Computerized Psychological Treatments for Adult Depression: A Meta-Analysis. Cogn. Behav. Ther..

[52] Racine N., McArthur B.A., Cooke J.E., Eirich R., Zhu J., Madigan S. (2021). Global Prevalence of Depressive and Anxiety Symptoms in Children and Adolescents During COVID-19: A Meta-Analysis. JAMA Pediatr..

[53] Werner-Seidler A., Perry Y., Calear A.L., Newby J.M., Christensen H. (2016). School-Based Depression and Anxiety Prevention Programs for Young People: A Systematic Review and Meta-Analysis. Clin. Psychol. Rev..

[54] Weisz J.R., Kuppens S., Ng M.Y., Eckshtain D., Ugueto A.M., Vaughn-Coaxum R., Jensen-Doss A., Hawley K.M., Krumholz Marchette L.S., Chu B.C. (2017). What Five Decades of Research Tells Us about the Effects of Youth Psychological Therapy: A Multilevel Meta-Analysis and Implications for Science and Practice. Am. Psychol..

[55] Skivington K., Matthews L., Simpson S.A., Craig P., Baird J., Blazeby J.M., Boyd K.A., Craig N., French D.P., McIntosh E. (2021). A New Framework for Developing and Evaluating Complex Interventions: Update of Medical Research Council Guidance. BMJ.

[56] Polanczyk G.V., Salum G.A., Sugaya L.S., Caye A., Rohde L.A. (2015). Annual Research Review: A Meta-analysis of the Worldwide Prevalence of Mental Disorders in Children and Adolescents. Child Psychol. Psychiatry.

[57] Solmi M., Radua J., Olivola M., Croce E., Soardo L., Salazar De Pablo G., Il Shin J., Kirkbride J.B., Jones P., Kim J.H. (2022). Age at Onset of Mental Disorders Worldwide: Large-Scale Meta-Analysis of 192 Epidemiological Studies. Mol. Psychiatry.

[58] Weiner B.J., Lewis C.C., Stanick C., Powell B.J., Dorsey C.N., Clary A.S., Boynton M.H., Halko H. (2017). Psychometric Assessment of Three Newly Developed Implementation Outcome Measures. Implement. Sci..

[59] Erskine H.E., Moffitt T.E., Copeland W.E., Costello E.J., Ferrari A.J., Patton G., Degenhardt L., Vos T., Whiteford H.A., Scott J.G. (2015). A Heavy Burden on Young Minds: The Global Burden of Mental and Substance Use Disorders in Children and Youth. Psychol. Med..

[60] Taylor R.D., Oberle E., Durlak J.A., Weissberg R.P. (2017). Promoting Positive Youth Development Through School-Based Social and Emotional Learning Interventions: A Meta-Analysis of Follow-Up Effects. Child Dev..

[61] Liverpool S., Mota C.P., Sales C.M.D., Čuš A., Carletto S., Hancheva C., Sousa S., Cerón S.C., Moreno-Peral P., Pietrabissa G. (2020). Engaging Children and Young People in Digital Mental Health Interventions: Systematic Review of Modes of Delivery, Facilitators, and Barriers. J. Med. Internet Res..

[62] Schleider J., Weisz J. (2018). A Single-session Growth Mindset Intervention for Adolescent Anxiety and Depression: 9-month Outcomes of a Randomized Trial. Child Psychol. Psychiatry.

[63] Schleider J.L., Weisz J.R. (2017). Little Treatments, Promising Effects? Meta-Analysis of Single-Session Interventions for Youth Psychiatric Problems. J. Am. Acad. Child Adolesc. Psychiatry.

[64] Hammen C. (2005). Stress and Depression. Annu. Rev. Clin. Psychol..

[65] Gotlib I.H., Joormann J. (2010). Cognition and Depression: Current Status and Future Directions. Annu. Rev. Clin. Psychol..

[66] Kessler R.C., Bromet E.J. (2013). The Epidemiology of Depression Across Cultures. Annu. Rev. Public Health.

[67] Smith M.V., Mazure C.M. (2021). Mental Health and Wealth: Depression, Gender, Poverty, and Parenting. Annu. Rev. Clin. Psychol..

[68] Shek D.T.L., Dou D. (2024). The Reach and Impact of a Positive Youth Development Program (Project P.A.T.H.S.) in China and Beyond: Review and Reflection. Appl. Res. Qual. Life.

[69] Hoagwood K., Vincent A., Acri M., Morrissey M., Seibel L., Guo F., Flores C., Seag D., Peth Pierce R., Horwitz S. (2022). Reducing Anxiety and Stress among Youth in a CBT-Based Equine-Assisted Adaptive Riding Program. Animals.

[70] Uhlig S., Jansen E., Scherder E. (2016). Study Protocol RapMusicTherapy for Emotion Regulation in a School Setting. Psychol. Music.

[71] Bowers R.M., Bowers E.P. (2023). A Literature Review on the Role of Hope in Promoting Positive Youth Development across Non-WEIRD Contexts. Children.

[72] Huang X. (2023). Writing with an Accent: Travelling Scholars and Xenophone Scholarship. Eur. J. Women’s Stud..

[73] Alatas S.F. (2003). Academic Dependency and the Global Division of Labour in the Social Sciences. Curr. Sociol..

[74] Go J. (2020). Race, Empire, and Epistemic Exclusion: Or the Structures of Sociological Thought. Sociol. Theory.

[75] Shek D.T.L., Yu L., Fu X. (2013). Confucian Virtues and Chinese Adolescent Development: A Conceptual Review. Int. J. Adolesc. Med. Health.

[76] Shek D.T.L., Ma C.M.S. (2012). Subjective Outcome Evaluation of the Project P.A.T.H.S. in Different Cohorts of Students. Sci. World J..

[77] Ma C.M.S., Shek D.T.L., Chen J.M.T. (2019). Changes in the Participants in a Community-Based Positive Youth Development Program in Hong Kong: Objective Outcome Evaluation Using a One-Group Pretest-Posttest Design. Appl. Res. Qual. Life.

[78] Zhu X., Shek D.T.L. (2020). Impact of a Positive Youth Development Program on Junior High School Students in Mainland China: A Pioneer Study. Child. Youth Serv. Rev..

[79] Zhou Z., Mu L., Qi S., Shek D.T.L. (2022). Service Leadership through Serving Minority Adolescents in Rural China Using a Rural Version of a Positive Youth Development Program. Appl. Res. Qual. Life.

[80] Catalano R.F., Fagan A.A., Gavin L.E., Greenberg M.T., Irwin C.E., Ross D.A., Shek D.T. (2012). Worldwide Application of Prevention Science in Adolescent Health. Lancet.

[81] Bachmann C.J., Scholle O., Bliddal M., Dosreis S., Odsbu I., Skurtveit S., Wesselhoeft R., Vivirito A., Zhang C., Scott S. (2024). Recognition and Management of Children and Adolescents with Conduct Disorder: A Real-World Data Study from Four Western Countries. Child Adolesc. Psychiatry Ment. Health.

[82] Bruckauf Z., Walsh S.D. (2018). Adolescents’ Multiple and Individual Risk Behaviors: Examining the Link with Excessive Sugar Consumption across 26 Industrialized Countries. Soc. Sci. Med..

[83] Campbell O.L.K., Bann D., Patalay P. (2021). The Gender Gap in Adolescent Mental Health: A Cross-National Investigation of 566,829 Adolescents across 73 Countries. SSM Popul. Health.

[84] Andersson G. (2018). Internet Interventions: Past, Present and Future. Internet Interv..

[85] Rauschenberg C., Schick A., Hirjak D., Seidler A., Paetzold I., Apfelbacher C., Riedel-Heller S.G., Reininghaus U. (2021). Evidence Synthesis of Digital Interventions to Mitigate the Negative Impact of the COVID-19 Pandemic on Public Mental Health: Rapid Meta-Review. J. Med. Internet Res..

[86] Rauschenberg C., Schick A., Goetzl C., Roehr S., Riedel-Heller S.G., Koppe G., Durstewitz D., Krumm S., Reininghaus U. (2021). Social Isolation, Mental Health, and Use of Digital Interventions in Youth during the COVID-19 Pandemic: A Nationally Representative Survey. Eur. Psychiatr..

[87] Yosep I., Hikmat R., Mardhiyah A. (2023). Types of Digital-Based Nursing Interventions for Reducing Stress and Depression Symptoms on Adolescents During COVID-19 Pandemic: A Scoping Review. J. Multidiscip. Healthc..

[88] Dwivedi Y.K., Kshetri N., Hughes L., Slade E.L., Jeyaraj A., Kar A.K., Baabdullah A.M., Koohang A., Raghavan V., Ahuja M. (2023). Opinion Paper: “So What If ChatGPT Wrote It?” Multidisciplinary Perspectives on Opportunities, Challenges and Implications of Generative Conversational AI for Research, Practice and Policy. Int. J. Inf. Manag..

[89] King D.R., Nanda G., Stoddard J., Dempsey A., Hergert S., Shore J.H., Torous J. (2023). An Introduction to Generative Artificial Intelligence in Mental Health Care: Considerations and Guidance. Curr. Psychiatry Rep..

[90] Han S.P., Kumwenda B. (2024). Bridging the Digital Divide: Promoting Equal Access to Online Learning for Health Professions in an Unequal World. Med. Educ..

[91] Albano A.M., Kendall P.C. (2002). Cognitive Behavioural Therapy for Children and Adolescents with Anxiety Disorders: Clinical Research Advances. Int. Rev. Psychiatry.

[92] James A.C., James G., Cowdrey F.A., Soler A., Choke A. (2015). Cognitive Behavioural Therapy for Anxiety Disorders in Children and Adolescents. Cochrane Database Syst. Rev..

[93] Reid J.E., Laws K.R., Drummond L., Vismara M., Grancini B., Mpavaenda D., Fineberg N.A. (2021). Cognitive Behavioural Therapy with Exposure and Response Prevention in the Treatment of Obsessive-Compulsive Disorder: A Systematic Review and Meta-Analysis of Randomised Controlled Trials. Compr. Psychiatry.

[94] Luxton R., Kyriakopoulos M. (2019). Depression in Children and Young People: Identification and Management. Arch. Dis. Child.-Educ. Pract..

[95] Khan K., Hall C.L., Babbage C., Dodzo S., Greenhalgh C., Lucassen M., Merry S., Sayal K., Sprange K., Stasiak K. (2024). Precision Computerised Cognitive Behavioural Therapy (cCBT) for Adolescents with Depression: A Pilot and Feasibility Randomised Controlled Trial Protocol for SPARX-UK. Pilot Feasibility Stud..

[96] Richards D.A., Ekers D., McMillan D., Taylor R.S., Byford S., Warren F.C., Barrett B., Farrand P.A., Gilbody S., Kuyken W. (2016). Cost and Outcome of Behavioural Activation versus Cognitive Behavioural Therapy for Depression (COBRA): A Randomised, Controlled, Non-Inferiority Trial. Lancet.

[97] Hinton D.E., Patel A. (2017). Cultural Adaptations of Cognitive Behavioral Therapy. Psychiatr. Clin. N. Am..

[98] Ciocanel O., Power K., Eriksen A., Gillings K. (2017). Effectiveness of Positive Youth Development Interventions: A Meta-Analysis of Randomized Controlled Trials. J. Youth Adolesc..

[99] Shek D.T., Dou D., Zhu X., Chai W. (2019). Positive Youth Development: Current Perspectives. Adolesc. Health Med. Ther..

[100] Yang W. (2024). Evidence-Based Social Science: Why, What, and Future Implications. Humanit. Soc. Sci. Commun..

[101] Raitskaya L., Tikhonova E. (2024). Evidence-Based Social Sciences and Practices: A Scoping Review. J. Lang. Educ..

[102] Kratochwill T.R., Shernoff E.S. (2003). Evidence-Based Practice: Promoting Evidence-Based Interventions in School Psychology. Sch. Psychol. Rev..

[103] Smith K.A., Ostinelli E.G., Ede R., Allard L., Thomson M., Hewitt K., Brown P., Zangani C., Jenkins M., Hinze V. (2023). Assessing the Impact of Evidence-Based Mental Health Guidance During the COVID-19 Pandemic: Systematic Review and Qualitative Evaluation. JMIR Ment. Health.

[104] Stein B.D., Jaycox L.H., Kataoka S.H., Wong M., Tu W., Elliott M.N., Fink A. (2003). A Mental Health Intervention for Schoolchildren Exposed to Violence: A Randomized Controlled Trial. JAMA.

[105] Dunning D.L., Griffiths K., Kuyken W., Crane C., Foulkes L., Parker J., Dalgleish T. (2019). Research Review: The Effects of Mindfulness-based Interventions on Cognition and Mental Health in Children and Adolescents—A Meta-analysis of Randomized Controlled Trials. Child Psychol. Psychiatry.

[106] Zhou Y., Duan Y., Zhou J., Qin N., Liu X., Kang Y., Wan Z., Zhou X., Li Y., Luo J. (2024). Character Strength-Based Cognitive-Behavioral Therapy Focusing on Adolescent and Young Adult Cancer Patients with Distress: A Randomized Control Trial of Positive Psychology. J. Happiness Stud..

[107] Hagger M. (2022). Meta-Analysis. Int. Rev. Sport Exerc. Psychol..

[108] Sigman M. (2011). A Meta-Analysis of Meta-Analyses. Fertil. Steril..

[109] Des Jarlais D.C., Lyles C., Crepaz N., the TREND Group (2004). Improving the Reporting Quality of Nonrandomized Evaluations of Behavioral and Public Health Interventions: The TREND Statement. Am. J. Public Health.

[110] Shek D.T.L. (2024). Enhancement of Psychosocial Competence and Well-being of Chinese High School Students under the COVID-19 Pandemic: Tin Ka Ping P.A.T.H.S. Project in Mainland China. Appl. Res. Qual. Life.

